# Better Diffusion Segmentation in Acute Ischemic Stroke Through Automatic Tree Learning Anomaly Segmentation

**DOI:** 10.3389/fninf.2018.00021

**Published:** 2018-04-25

**Authors:** Jens K. Boldsen, Thorbjørn S. Engedal, Salvador Pedraza, Tae-Hee Cho, Götz Thomalla, Norbert Nighoghossian, Jean-Claude Baron, Jens Fiehler, Leif Østergaard, Kim Mouridsen

**Affiliations:** ^1^Department of Clinical Medicine, Center of Functional Integrative Neuroscience, Aarhus University, Aarhus, Denmark; ^2^Radiology Department, IDI, Hospital Dr Josep Trueta, Institut d'Investigació Biomèdica de Girona (IDIBGI), University of Girona, Girona, Spain; ^3^Stroke Medicine Department, Hôpital Neurologique, Hospices Civils de Lyon, Lyon, France; ^4^Creatis, Centre National de la Recherche Scientifique UMR 5220, Institut National de la Santé et de la Recherche Médicale U1206, INSA de Lyon, Université Lyon 1, Lyon, France; ^5^Klinik und Poliklinik für Neurologie, Kopf- und Neurozentrum, Universitätsklinikum Hamburg-Eppendorf, Hamburg, Germany; ^6^Department of Neurology, Sainte-Anne Hôpital, Paris Descartes University, Institut National de la Santé et de la Recherche Médicale U894, Paris, France; ^7^Department of Clinical Neurosciences, University of Cambridge, Cambridge, United Kingdom; ^8^Department of Diagnostic and Interventional Neuroradiology, University Medical Center Hamburg-Eppendorf, Hamburg, Germany; ^9^Department of Neuroradiology, Aarhus University Hospital, Aarhus, Denmark

**Keywords:** stroke, diffusion MRI, segmentation, diffusion lesion, computer learning, decision trees

## Abstract

Stroke is the second most common cause of death worldwide, responsible for 6.24 million deaths in 2015 (about 11% of all deaths). Three out of four stroke survivors suffer long term disability, as many cannot return to their prior employment or live independently. Eighty-seven percent of strokes are ischemic. As an increasing volume of ischemic brain tissue proceeds to permanent infarction in the hours following the onset, immediate treatment is pivotal to increase the likelihood of good clinical outcome for the patient. Triaging stroke patients for active therapy requires assessment of the volume of salvageable and irreversible damaged tissue, respectively. With Magnetic Resonance Imaging (MRI), diffusion-weighted imaging is commonly used to assess the extent of permanently damaged tissue, the core lesion. To speed up and standardize decision-making in acute stroke management we present a fully automated algorithm, ATLAS, for delineating the core lesion. We compare performance to widely used threshold based methodology, as well as a recently proposed state-of-the-art algorithm: COMBAT Stroke. ATLAS is a machine learning algorithm trained to match the lesion delineation by human experts. The algorithm utilizes decision trees along with spatial pre- and post-regularization to outline the lesion. As input data the algorithm takes images from 108 patients with acute anterior circulation stroke from the I-Know multicenter study. We divided the data into training and test data using leave-one-out cross validation to assess performance in independent patients. Performance was quantified by the Dice index. The median Dice coefficient of ATLAS algorithm was 0.6122, which was significantly higher than COMBAT Stroke, with a median Dice coefficient of 0.5636 (*p* < 0.0001) and the best possible performing methods based on thresholding of the diffusion weighted images (median Dice coefficient: 0.3951) or the apparent diffusion coefficient (median Dice coefficeint: 0.2839). Furthermore, the volume of the ATLAS segmentation was compared to the volume of the expert segmentation, yielding a standard deviation of the residuals of 10.25 ml compared to 17.53 ml for COMBAT Stroke. Since accurate quantification of the volume of permanently damaged tissue is essential in acute stroke patients, ATLAS may contribute to more optimal patient triaging for active or supportive therapy.

## 1. Introduction

Magnetic Resonance Imaging (MRI) provides crucial information in the management of acute stroke patients (Barber et al., 1998). Diffusion weighted MRI (DWI) is sensitive to cellular water shifts following the breakdown of electrochemical membrane ion gradients after severe energy failure. Tissue that display increased image intensity on DWI images and reductions of the apparent diffusion coefficient (ADC) are generally thought to represent irreversibly damaged tissue, the so-called infarct core (Barber et al., 1998). The penumbra—the electrically silent, yet salvageable tissue—is the target of acute stroke therapy (Wheeler et al., 2013). When using MRI in acute stroke management, the penumbra is operationally defined as the mismatch between hypoperfused tissue, as determined by perfusion-weighted imaging (PWI), and the ischemic core, as determined by DWI (Barber et al., 1998; Schlaug et al., 1999). Often, PWI is bypassed to save precious time before treatment can be initiated, and salvageable tissue instead assessed by an operational “clinical/DWI” mismatch (Balami et al., 2013; Mishra et al., 2014). Patients who present with small diffusion lesions generally respond favorably to thrombolytic and endovascular therapy (Nagakane et al., 2011), while the odds of improving patient outcome decline as the diffusion lesion volume increases and the mismatch tissue volume declines. In particular, the balance between treatment success and risk of hemorrhagic side-effects may become unfavorable in patients with diffusion lesions above 70 ml (Sanak et al., 2006). There is hence a pressing need to develop fast, accurate, and reliable means of identifying core lesion on DWI images and thereby quantify their size, particularly as treatment windows for recanalization therapy are being extended by using tissue characteristics—so-called “tissue clocks”—rather than time from symptom onset—the traditional clock—to guide acute stroke therapy (Hillis and Baron, 2015).

Current methodologies for the identification of diffusion lesions either rely on fixed diffusion thresholds (Oppenheim et al., 2001; Sener, 2001; Straka et al., 2010; Purushotham et al., 2015), which may not be generally applicable across cohorts and scanner vendors, or they require the use of both diffusion and perfusion sequences (Nagenthiraja et al., 2013).

In order to identify the core lesion, a fast, accurate, and operator-independent automatic method would be desirable. Here we describe an adaptive, multimodal algorithm, ATLAS (Automatic Tree Learning Anomaly Segmentation), for swiftly identifying diffusion lesions. It combines DWI and ADC values to automatically identify diffusion lesions. We validate the technique in patient data from a multicenter study with *n* = 108 acute stroke patients, scanned with different protocols, field strengths, and system manufacturer, and then assess its performance relative to lesions detected by human experts.

## 2. Materials and methods

### 2.1. Patients and image acquisition

In all, 108 patients with anterior circulation strokes (67 male, 41 female) from the I-Know multicenter study were analyzed retrospectively (European Commission, 2006). The study conformed with the Helsinki Declaration, the rules laid out by the Council of Europe Convention on Human rights and Biomedicine, Directive 95/46/EC of the European Parliament and of the Council of 24 October 1995 on the protection of individuals with regard to the processing of personal data and on the free movement of such data, and with the legislation and regulations in Denmark, Germany, France, and Spain, respectively. The study were approved by the Aarhus, Hamburg, Lyon, and Girona hospitals respective regional ethics committees, and carried out after informed consent from the patients. All subjects gave written informed consent in accordance with the Declaration of Helsinki. Only patients with acute DWI scans were included. The median age of the patients was 70.5 years (range: 30, 92), the median time from onset of symptoms to initial MRI scan was 149 min (range: 46, 788), and the median NIHSS (National Institutes of Health Stroke Scale) was 11 (range: 4, 24) (Table 1).

**Table 1:** Pooled patient characteristics.

	**Median [range]**	***n***
Patients		108 (♀ = 41)
Age	70.5[30, 92]	
Time, Onset to MRI (minutes)	149[46, 788]	
NIHSS	11[4, 24]	
DWI volume (ml)	11.8[0, 164.8]	
Signal-to-noise rate DWI	13.1[4.5, 25.5]	
Stroke types		
*- Cardiac source of emboli*		51
*- Large vessel disease with significant carotid stenosis*		19
*- Large vessel disease, other*		13
*- Dissection*		3
*- Other/unusual cause*		1
*- Undetermined*		21
Visible occlusion		82
*- Intracranial ICA occlusion*		21
*- Carotid T occlusion*		1
*- M1 occlusion*		34
*- M2 occlusion*		29
*- MCA distal branch occlusion*		10
*- ACA occlusion*		2

Standard gradient echo dynamic susceptibility contrast MRI was performed on the scanner used for stroke MRI at the admitting hospital (GE Signa Excite 1.5*T*, GE Signa Excite 3*T*, GE Signa HDx 1.5*T*, GE Signa Horizon 1.5*T*, Milwaukee, WI; Siemens TrioTim 3*T*, Siemens Avanto 1.5*T*, Siemens Sonata 1.5*T*, Erlangen, Germany; Philips Gyroscan NT 1.5*T*, Phillips Achieva 1.5*T*, and Philips Intera 1.5*T*, Best, Netherlands). Echo-planar DWI was obtained at magnetic field gradient strengths of $b=0\frac{s}{mm^{2}}$ and $b=1000\frac{s}{mm^{2}}$, where the weighted images were acquired at 3–12 directions, according to the scanner vendor/type at the different centers. The data quality obtained across centers and scanner vendors is summarized in terms of image signal-to-noise ratios in Table 1.

### 2.2. Expert outlining of diffusion lesion

The core lesions were delineated on acute diffusion weighted images with adjunct ADC and T2FLAIR (T2 weighted Fluid Attenuated Inversion Recovery) images to avoid the effects of T2 shine through. Each core lesion was delineated by a single expert based on clinical experience. This delineated diffusion lesion was the goal of the segmentation algorithm.

### 2.3. Input to ATLAS

**Figure 1:**
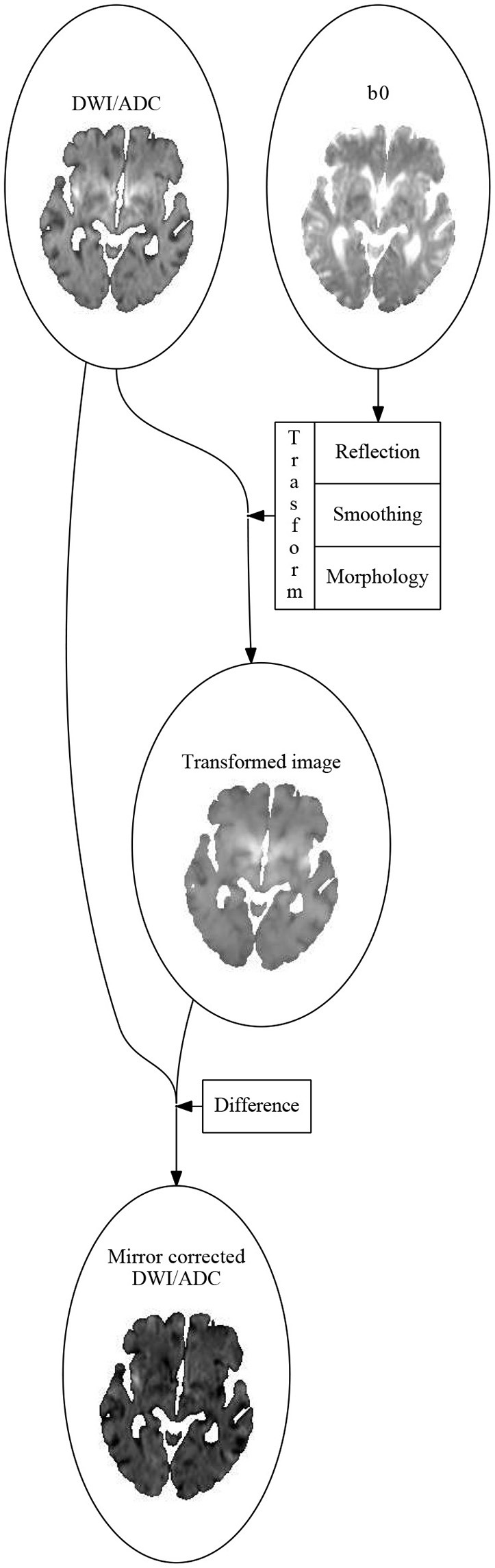
The algorithm for calculating the mirror correced images. First an affine transformation, sending the left hemisphere to the right hemisphere and vise versa, is calculated by coregitering the *b*_0_-image to the image you get by flipping it along the sagittal plane. The DWI (or ADC) image is then flipped using this transformation. The flipped image is smoothed using an 3-dimensional Gaussian isotropic kernel. The mirror corrected image is then made voxel for voxel by subtracting the current voxel value in the DWI (or ADC) image by the most critical (highest for DWI, lowest for ADC) value of the transformed and smoothed image in a small neighborhood around the current voxel.

ATLAS uses four parameters derived from the diffusion weighted sequence. These include the DWI image at $b=1000\frac{s}{mm^{2}}$ and the ADC map, which is uninfluenced by T2 effects due to edema, and quantifies water diffusion on an absolute scale. For comparison across patients, the DWI images were standardized by dividing image intensity values by the mean DWI value in the contralateral hemisphere for each axial slice in each patient. We employed one additional normalization step, utilizing contralateral mirror images to avoid normal structures, and artifacts being misinterpreted as lesions, as follows (see Figure 1):

For each image a transformation map was calculated. The transformation consisted of three parts:

1. The mirror image relative to the mid-sagittal plane, calculated by coregistering the *b*_0_ image to the *b*_0_ image flipped along the y-axis using SPM12 (Wellcome Trust Center for Neuroimaging, UCL, UK) (Friston et al., 2007).
2. A smoothing using an isotropic Gaussian kernel.
3. A morphological correction assigning to each voxel the intensity value of the voxels with the most critical intensity (high intensity for DWI, low for ADC) in a small neighborhood of the voxel.

The mirror corrected images were then the difference between the original image and the transformed image.

### 2.4. The ATLAS algorithm

The principle in the ATLAS algorithm is to build a deep decision tree, which, in an unconstrained fashion, identifies combinations of image marker values that optimally separate lesion and non-lesion voxels. Overfitting is typically reduced by a subsequent pruning step, which is based on the size of the tree itself, but we safeguarded the algorithm further by using the spatial relation between voxels in a second step.

### 2.5. Decision tree

The decision tree is built one node at a time, starting from the root and proceeding in a step-wise manner. For each variable, the threshold that yields the maximal Youden's Index (*J* = *sensitivity* + *specificity* − 1) is determined. The variable with the highest maximal Youden's Index is chosen, and the data is divided along the threshold that yields this maximal Youden's Index. Two branches are made from the node, one for the values greater than or equal to the threshold, and one for the values smaller than the threshold, and a new node (with the corresponding data) is made at the end of each of the two branches. This procedure is repeated until all the nodes in the bottom (the leaves) of the tree only have data points of one class—that is, either all data points at a leaf are inside the drawn lesion or all are outside the drawn lesion.

At each node *N*, we define the preliminary prediction value of the node as $p\left(N\right)=\frac{n_{1}+1}{n+2}$, where *n*_1_ is the number of in-lesion-voxels in the data of the node, and *n* is the total number of voxels in the data of the node. This value is chosen over the more obvious value $\frac{n_{1}}{n}$ based on the fact that the fewer data points left at a node, the more small errors and artifacts will skew the prediction. The value $\frac{n_{1}+1}{n+2}$, when compared to $\frac{n_{1}}{n}$, will be closer to 0.5 when there are few data points, and almost unchanged when there are many data points.

We have now built a decision tree. Any data point *x* can traverse the tree by starting at the root, and then following the path in the tree dictated by whether the value of *x* in the variable assigned to the current node is less than the threshold assigned to the node. We define *N*_*d*_(*x*) to be the node on the path of *x* in the tree reached after *d* steps. So *N*_0_(*x*) is the root of the tree, no matter the values of *x*. If *d* is longer than the full path of *x*, we just define *N*_*d*_(*x*) = *N*(*x*) to be the final node *x* will reach (the leaf). For each possible data point *x* we define preliminary predictions of *x* at depth *d* as *p*_*d*_(*x*) = *p*(*N*_*d*_(*x*)), and simply the preliminary predictions of *x* as *p*(*x*) = *p*(*N*(*x*)).

To avoid inflating small errors and artifacts, we include a pruning step. The pruning is done by finding an optimal depth of the tree (the maximal distance from the root to the leaves), and removing all nodes further away from the root than this depth. To find the optimal depth when building a tree based on *n* patients we first build *n* trees based *n* − 1 of the patients, where each patient is excluded from the building of one tree. The data points for each patient is now fed into the tree it was excluded from, to calculate all the *p*_*d*_(*x*) values. At each possible depth *d* we compute the quality of the prediction of the drawn lesion by the preliminary predictions at depth *d*, using the Area Under the Receiver Operating Curve (AUC) statistic. As the optimal depth of the full tree, we choose the median (over the set of patients) of the depths that yields the highest AUC value for the trees with one patient excluded.

### 2.6. Regularization

So far, the decision tree only utilizes spatial information in the limited fashion of the mirror corrected images. We therefore developed series of steps to regularize the decision tree lesion estimate. First, the probability map is smoothed with a 3-dimensional Gaussian isotropic kernel. Next, the smoothed image is thresholded at 0.25. This value is chosen due to the fact that there are many more voxels outside the lesion than inside, so the decision trees will naturally err to the side of voxels being outside lesions. Finally, the mask is morphologically closed, which has the effect of reducing false negative noise, and then morphologically opened which has the effect of reducing false positive noise.

### 2.7. Evaluation

As a measure of how well segmentation was performed, we use the Dice coefficient, which is twice the overlapping volume (or the true positives, *TP*) divided by the sum of the volume of the prediction (true positive + false positive, *TP* + *FP*) and the volume of the actual segment (true positive + false negative, *TP* + *FN*), or $D=\frac{2TP}{2TP+FP+FN}$. Note that this yields a number between 0 and 1, where 0 means that the actual and predicted lesions did not overlap while 1 means that the actual and predicted lesions were exactly equal.

To validate the method, we used leave-one-out cross-validation to build trees based on all but one patient. This model was then evaluated on the patient that was left out.

We compared the results to both the existing COMBAT Stroke method (Nagenthiraja et al., 2013) and to a generalized thresholds-based method.

#### 2.7.1. Generalized threshold

Thresholding is the simplest and fastest ways of making a segmentations, and therefore appealing (Oppenheim et al., 2001; Sener, 2001; Purushotham et al., 2015). Thresholding methods work by including all voxels with an intensity greater or smaller than a certain value into the lesion. Often single voxels with very high intensities or very low intensities are removed beforehand due to them being deemed to be noise or artifacts rather than stroke lesions. The result of this is that instead of one threshold the methods uses two thresholds, choosing a segment of all the voxels with intensitied between two given values. When evaluating the ATLAS algorithm we compare our results to the results of optimal generalized thresholding on both ADC and DWI. This optimal generalized thresholding works by finding the two thresholds yielding the higest Dice coefficient for each patient. We emphasize that since the thresholds are optimized for each individual patient, these optimal per-patient performance estimates are not applicable in prospective patients but serve here to benchmark ATLAS against a best-case scenario, that is, they represent upper-most performance bounds for methods that apply up to two thresholds to ADC or DWI images, respectively.

## 3. Results

**Figure 2:**
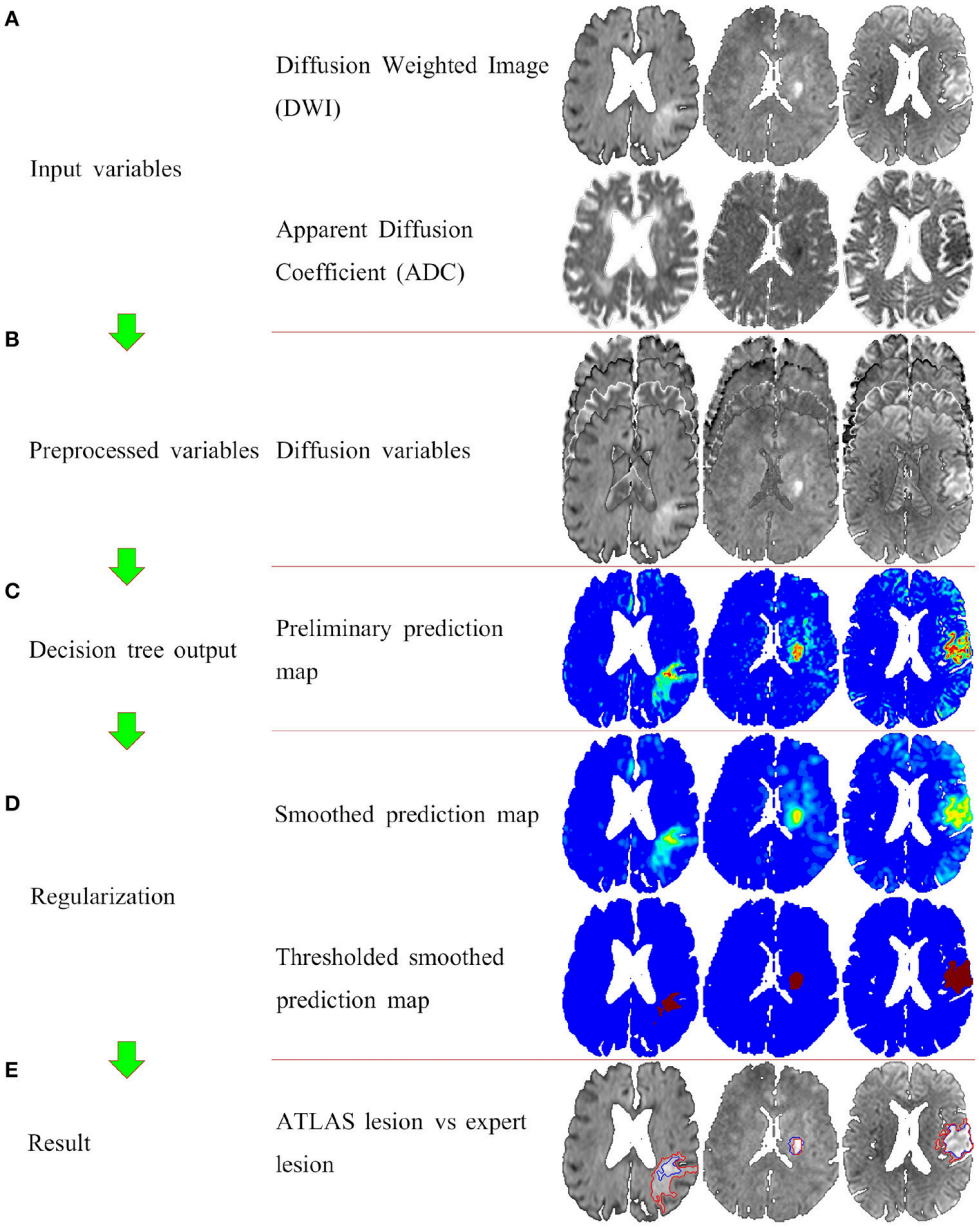
The full ATLAS algorithm with examples of intermediate results. Panel **(A)** shows the input variables (the DWI and the ADC images). Panel **(B)** shows the preprocessed variables, that is the original two variable, and the mirror corrected versions of the original two variables. Panel **(C)** illustrated the voxelvise output of the decision tree. Panel **(D)** shows the postprocessing of first smoothing the output, and then thresholding. Finally panel **(E)** shows the ATLAS segmentation (blue outline) overlaying the original DWI image along with the expert drawn segmentation (red outline).

Figure 2 shows the segmentation process for three similar slices from three different patients with Dice values closest to the first, second, and third quartile. Figure 2A shows the two input variables used: DWI, ADC. Figure 2B shows the variables that are put into the decision tree i.e., DWI, ADC, mirror corrected DWI, and mirror corrected ADC. With this input the decision tree now provides us with Figure 2C: the preliminary prediction map. In Figure 2D, the first prediction map is smoothed with a 3-dimensional Gaussian kernel and then thresholded at 0.25. Finally, the result (blue contour) is compared to the expert result (red outline) in Figure 2E.

**Figure 3:**
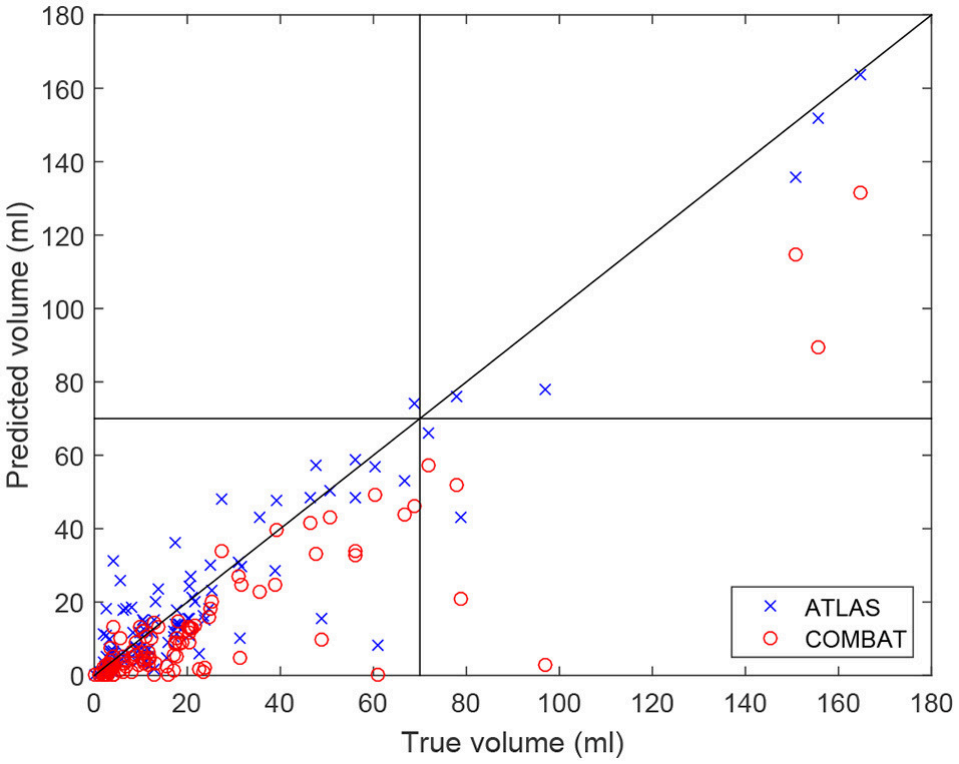
The predicted volumes of the lesions segmented by the ATLAS algorithm and by the Combat stroke method compared to the volume segmented by the expert outlining.

In Figure 3, the volume of the lesion mask drawn by the experts is compared to the volume of the prediction mask as determined by the ATLAS algorithm and COMBAT Stroke diffusion lesion segmentation, respectively (Nagenthiraja et al., 2013). The identity line indicates a perfect match between predicted volume and true volume. For the ATLAS prediction, we observe a standard deviation of the residuals of 10.25 ml compared to 17.53 ml for COMBAT Stroke. The horizontal and vertical lines at 70 ml indicate the volumes above which thrombolysis is discouraged according to current guidelines (Sanak et al., 2006).

**Figure 4:**
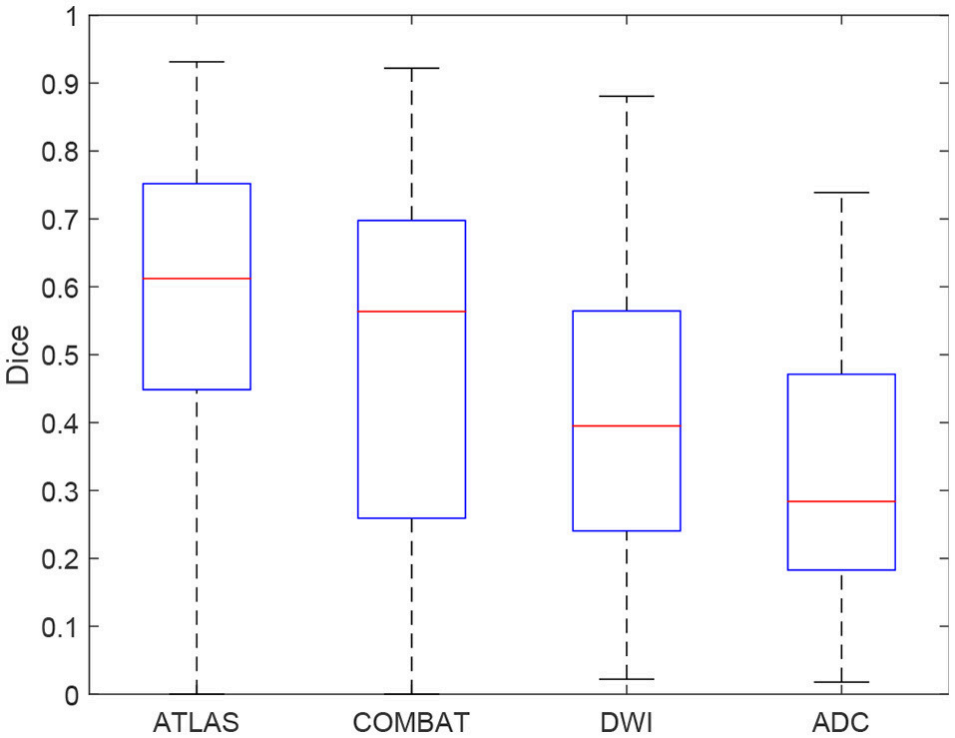
A boxplot of the Dice coefficients for the ATLAS segmentation, the Combat stroke segmentation and the best possible solely threshold based segmentation by DWI and by ADC.

In Figure 4, individual Dice coefficients are compared for lesion predictions by ATLAS, COMBAT Stroke, and generalized thresholds on the DWI image and on the ADC image. ATLAS yields Dice coefficients with median 0.6122 and an interquartile range of [0.4486, 0.7519], COMBAT Stroke yields Dice coefficients with median 0.5636 and an interquartile range of [0.2592, 0.6977], DWI yields Dice coefficients with median 0.3951 and an interquartile range of [0.2405, 0.5644] and ADC yields Dice coefficients with median 0.2839 and an interquartile range of [0.1828, 0.4712]. The ATLAS Dice coefficients median is significantly larger than that of Combat Stroke (Wilcoxon signed-rank test, *p* = 1.9·10^−6^).

## 4. Discussion

We have presented a novel, automated, algorithm for identifying lesions on DWI images obtained from acute ischemic stroke patients. The algorithm does not require other acquisitions than the acute diffusion scan and is devised to produce outlines of diffusion lesions that agree with human experts. Our results show that lesions identified by the ATLAS algorithm are in good agreement with the lesions identified by human experts, both with regard to their volume and with regard to their localization. Even in cases where the classification based on ATLAS differed from that of the expert drawn lesions, the actual difference in volume was modest. The ATLAS algorithm outperforms other methods such as the COMBAT Stroke method, and substantially outperforms the optimized thresholding approaches, such as the RAPID (Straka et al., 2010). The algorithm provides a more adaptive approach than segmentation of image according to a prespecified image threshold.

The ATLAS model takes a long time to train, especially in terms of building the decision tree, the subsequent tree-search and probability map/lesion volume calculations are fast. Accordingly, the most time-consuming part of this calculation is the preparation of mirror corrected maps, which can be achieved in seconds on an ordinary computer. Our results indicate that threshold-based segmentation methods, such as RAPID, are less precise and reliable than ATLAS or COMBAT Stroke. In particular, the RAPID method requires manual removal of artefacts, which makes it much more time consuming, more subjective, and thus less reproducible. While the COMBAT Stroke method does almost as well as ATLAS, it requires perfusion MRI data.

### 4.1. Limitations to the study

The study is limited by its retrospective nature. The I-Know database only contains anterior circulation strokes. Although they represent the majority of stroke cases, this means that it is not really a study about acute stroke in general, but rather a study about acute anterior circulation strokes. Furthermore, the I-Know database is a multicenter database. This leads to greater variation, due to the variation in scanners, field strenghts, number of head coil channels, and clinical practices. This can both be an advantage in the sense of yielding a more robust model—one working with more varied data, but it also requires more data to build the more stable model. Though this study included a very large number of data points (~16, 400, 000 voxels), the variation, due to the data being from a multicenter database, is patientwise, and 108 patients is still a low number of patients to fully capture the variability of stroke lesions as they appear on diffusion weighted images. The best solution to these limitations is to include more data in the building of the model.

The manual outlining of the lesion is another limitation. The goal was to segment the ischemic core, but the model is trained to find the DWI lesion—an experts best estimation of the ischemic core. This means that common and systematic errors in this expert estimation may be included in the model (Ay et al., 2008; Campbell et al., 2010). To remedy this one can try to reduce the number of errors by using more experts, and to get a consensus DWI lesion. While this may improve the quality of the data, there is so far no method of avoiding the problem.

## 5. Conclusion

By providing diffusion lesion volume estimates, the algorithm provides reliable guidance to clinicians as they weigh the potential benefits of administering thrombolytic therapy. The ATLAS algorithm segments the diffusion lesion in a fully automatic way, while outperforming state-of-the-art methods and any possible methods solely base on thresholds of the DWI or the ADC values.

## Author contributions

JB got the idea, did the analysis and wrote the manuscript. TE assisted in the writing process, especially the more medical parts and did a critical review. SP, T-HC, GT, NN, J-CB, JF, and LØ did data acquisition and critical review. KM assisted in the writing process and critical review.

### Conflict of interest statement

KM is a shareholder in COMBAT Stroke ApS. The other authors declare that the research was conducted in the absence of any commercial or financial relationships that could be construed as a potential conflict of interest.
